# Sarcopenia and Sarcopenic Obesity in Patients with Muscular Dystrophy

**DOI:** 10.3389/fnagi.2014.00274

**Published:** 2014-10-07

**Authors:** Luciano Merlini, Alessandro Vagheggini, Daniela Cocchi

**Affiliations:** ^1^Laboratory of Musculoskeletal Cell Biology, Istituto Ortopedico Rizzoli, Bologna, Italy; ^2^Department of Statistical Sciences, University of Bologna, Bologna, Italy

**Keywords:** muscular dystrophy, sarcopenia, sarcopenic obesity, body composition, muscle strength, physical performance

## Abstract

Aging sarcopenia and muscular dystrophy (MD) are two conditions characterized by lower skeletal muscle quantity, lower muscle strength, and lower physical performance. Aging is associated with a peculiar alteration in body composition called “sarcopenic obesity” characterized by a decrease in lean body mass and increase in fat mass. To evaluate the presence of sarcopenia and obesity in a cohort of adult patients with MD, we have used the measurement techniques considered golden standard for sarcopenia that is for muscle mass dual-energy X-ray absorptiometry (DXA), for muscle strength hand-held dynamometry (HHD), and for physical performance gait speed. The study involved 14 adult patients with different types of MD. We were able to demonstrate that all patients were sarcopenic obese. We showed, in fact, that all were sarcopenic based on appendicular lean, fat and bone free, mass index (ALMI). In addition, all resulted obese according to the percentage of body fat determined by DXA in contrast to their body mass index ranging from underweight to obese. Skeletal muscle mass determined by DXA was markedly reduced in all patients and correlated with residual muscle strength determined by HHD, and physical performances determined by gait speed and respiratory function. Finally, we showed that ALMI was the best linear explicator of muscle strength and physical function. Altogether, our study suggests the relevance of a proper evaluation of body composition in MD and we propose to use, both in research and practice, the measurement techniques that has already been demonstrated effective in aging sarcopenia.

## Introduction

Muscular dystrophies (MDs) are a clinically and genetically heterogeneous group of inherited diseases characterized by progressive muscle wasting and weakness (Emery, 2002). In the dystrophic muscle, there is a progressive loss of muscle fibers that are substituted by fat and connective tissue. The dystrophic muscle weakness reflects the loss of muscle tissue and compromises physical performance. In summary, MD can specifically be defined as a condition characterized by lower skeletal muscle quantity, lower muscle strength, and lower physical performance. The other condition that combines these three characteristics is sarcopenia.

Sarcopenia is defined according to European consensus group (Cruz-Jentoft et al., 2010) as a condition that involves a loss of muscle mass and declining strength and/or physical performance. In particular, “severe sarcopenia” is the stage identified when all three criteria of the definition are met (low muscle mass, low muscle strength, and low physical performance) (Cruz-Jentoft et al., 2010). With aging, skeletal muscle atrophy in human beings appears to be inevitable. A gradual loss of muscle fibers begins at approximately 50 years of age and continues such that by 80 years of age, approximately 50% of the fibers are lost from the limb muscles that have been studied (Faulkner et al., 2007). Although primarily a disease of the elderly, sarcopenia can be observed at any age resulting associated with conditions like disuse, malnutrition, and neuromuscular diseases (Muscaritoli et al., 2010).

Body composition changes with aging. Cross-sectional and longitudinal data have shown that aging is associated with a peculiar alteration in body composition – a decrease in lean body mass and increase in fat mass (FM). Baumgartner et al. (2004) have proposed criteria for this new condition that combines sarcopenia and obesity, which has been called sarcopenic/obesity (Baumgartner et al., 2004). High body mass index (BMI), low muscle mass, and the combination of excess FM with low muscle mass have been associated with increased risk for physical disability in older adults (Fielding et al., 2011). It is, therefore, recognized that sarcopenic/obesity has an important impact on physical capacity in older individuals as the prevalence of this condition is <10% of the elderly population (Baumgartner et al., 2004; Batsis et al., 2013), whereas the prevalence of sarcopenia is 13–36% and circa 30% of older Americans are obese (Jankowski et al., 2008).

The aim of this study was to evaluate the presence and severity of sarcopenia and of sarcopenic obesity in a cohort of adult patients with MD. In order to conduct a valid and reliable diagnosis of these conditions in patients with MD, we have measured muscle mass, muscle strength, and physical performance. The measurement techniques were the ones suggested by the European Working Group, recognized useful both in research and practice that is for muscle mass dual-energy X-ray absorptiometry (DXA), for muscle strength hand-held dynamometry (HHD), and for physical performance gait speed (Mijnarends et al., 2013).

## Materials and Methods

### Patients

We included in the study, adult patients with a clinical and molecular diagnosis of MD and clinical and body composition evaluations performed within 1 month and with the same equipment. Data of 14 adult patients (7 were women) with a clinical/laboratory phenotype compatible with the diagnosis of MD were collected. The mean age of women was 35.6 (range 19–56) years and of men 29.5 (range 18–64) years; this difference was not significant (*p* = 0.47). All the patients had a definite molecular diagnosis. There were six patients with Bethlem myopathy (BM) and one with Ullrich congenital muscular dystrophy (UCMD) with recognition of a pathogenetic mutation/s in one of the three *COL6* genes (Pepe et al., 1999; Lucioli et al., 2005; Merlini et al., 2008b; Gualandi et al., 2009, 2011; Martoni et al., 2009; Sabatelli et al., 2011, 2012). Two patients had a rigid spine syndrome due to a mutation of the FHL1 gene. One patient had a limb girdle MD type 2D (LGMD2D) due to a homozygous mutation in the alpha-sarcoglycan gene. Three patients had a Duchenne MD (DMD) and one a Becker MD (BMD) with mutations in the *DMD* gene. Three patients were non-ambulatory: the UCMD patient was never able to walk and was on nocturnal non-invasive ventilation, the patient with LGMD2D and one with rigid spine lost ambulation at 15 and 21 years of age, respectively. The study protocol was approved for patients with MD by the institutional ethical committee in 1994 and specifically for patients with COL6 related myopathies in 2011 (ClinicalTrials.gov identifier: NCT01438788). All subjects were fully informed and gave their written informed consent.

### Body composition

Body composition was obtained by DXA (Hologic 4500 W; software version 11.2; Hologic, Inc., Waltham, MA). According to the tree-compartment model of body composition, the Hologic software provided regional and whole body estimation of lean mass (LM), FM, and bone mineral content (BMC). BMC and LM were added to obtain fat-free mass (FFM). Appendicular lean mass (ALM) was the sum of bone-free and fat-free tissue mass in the arms and legs. Body mass, FM, LM, FFM, and ALM were normalized to height^2^ to control for skeletal size. ALM/height^2^, appendicular lean mass index (ALMI), was considered the sarcopenia index. ALMI two standard deviations (Baumgartner et al., 1998) or more (Tanko et al., 2002; Newman et al., 2003) lower than a mean derived from young male and female reference groups was defined as the gender-specific cut point for sarcopenia (Baumgartner et al., 1998; Tanko et al., 2002; Newman et al., 2003). BMI, an index of obesity, was derived from body mass measured by DXA to the nearest gram and height measured to the nearest 0.1 cm. We used BMI to categorized participants as obese (BMI ≥ 30), overweight (25 ≤ BMI < 30), normal weight (18.5 < BMI < 25), or underweight (≤18.5) (WHO, 2000). Sarcopenic obesity was defined according to Baumgartner et al. (2004) as ALMI < 7.26 kg/m^2^ in men and 5.45 kg/m^2^ in women and percentage body fat greater than 28% in men and 40% in women (Baumgartner et al., 2004).

### Muscle strength and physical performances

Maximal isometric strength was assessed using a hand-held dynamometer (Type CT 3001, Citec, C.I.T. Technics BV, Groningen, The Netherlands) (van der Ploeg et al., 1991). Two muscle groups were examined bilaterally: handgrip (HG) and knee extensor (KE) (Merlini et al., 2002, 2003, 2004). The maximum force from three attempts was used in analysis. Patients were considered to have low muscle strength if HG strength was <20 kg in women and <30 kg in men (Bohannon et al., 2006; Cruz-Jentoft et al., 2010). This is also the diagnostic threshold in handgrip strength that best discriminates subjects with mobility limitations to be used in clinical practice (Fried et al., 2001; Lauretani et al., 2003). A ratio of maximal muscular strength of knee extensors to body weight was calculated by dividing the muscular strength (N) by body weight (kg). In older adults (81–89 years), muscular strength thresholds to perform activities of daily living (ADL) independently were 2.8 N/kg for knee extensors (Hasegawa et al., 2008). Ploutz-Snyder et al. (2002) have found similar figures: ambulatory tasks (chair rise, gait speed, and stair ascent and descent) were compromised in individuals with a ratio of isometric leg extension peak torque to body weight (STR/WT) <3.0 N m/kg (Ploutz-Snyder et al., 2002).

Forced vital capacity (FVC) was determined with an electronic spirometer, and percent-predicted values were calculated based on normal published values. A value between 50 and 70% was considered moderately reduced; a value less than 50% was considered severely reduced (van der Kooi et al., 2006). Timed test included the time to walk 10 m. Patients were considered to have a low mobility based on the walking speed threshold of 1.22 m/s proposed by Langlois et al. (1997), evaluating the ability to cross the street in the time typically allotted at signalized intersections (Langlois et al., 1997).

### Statistical analysis

In order to determine the separate relative contributions of the various indices to the strength variables, we used the linear regression models. Due to the limited sample size, we chose to estimate the linear regression model without intercept, *y*_i_ = *bx*_i_ + *e*_i_, via ordinary least squares. Each *b* coefficient is the slope of the regression line and represents the increment/decrement of the dependent variable for a unit increment of each explanatory variable. Linear correlation coefficients have, therefore, been computed for various couples of variables with the aim of choosing the variables for simple linear models without intercept. High values of the linear correlation coefficients helped in the choice of identifying the body mass and muscularity as explanatory variables to the dependent variables muscle strength, gait speed, and pulmonary function. The strength of each simple linear relationship was investigated via the adjusted *R*^2^, which represents a well-known goodness of fit measure. Measurable variables are presented as mean (*x*) ± SD and their range of variation is presented in parentheses.

## Results

### Body composition

Six participants were normal weight, four overweight, three underweight, and one obese based on BMI (Table 1). All were sarcopenic based on ALMI, which was well below of the cut off for the sarcopenia index of 7.26 kg/m^2^ for men and 5.45 for women, and all were sarcopenic obese based on ALMI and % FM that was greater than 28% in men and 40% in women. In addition, ALMI was significantly different (*p* ≈ 0.00) between walkers and non-walkers (4.6 ± 0.8 vs. 3.4 ± 0.3). FFMI was well below the fifth percentile for all the patients as compared to the normal age-related Italian population and also to the 70–80–year olds (17 kg/m^2^ in men and 13.4 kg/m^2^ in women) (Coin et al., 2008). Gender differences in body composition were significant for the indices of muscularity (FFMI, LMI, and ALMI) but not for total mass, BMI, and FMI.

**Table 1:** **Body mass, adiposity, and muscularity**.

	Women (*n* = 7)	Men (*n* = 7)	*p*-value
Total mass (kg)	60.90 ± 13.09 (45.47–76.38)	61.05 ± 16.80 (40.01–79.63)	0.99
BMI (kg/m^2^)	22.41 ± 4.61 (16.10–29.20)	23.99 ± 4.79 (17.40–31.70)	0.54
Total fat (%)	50.66 ± 6.02 (41.30–58.50)	42.93 ± 8.61 (29.70–58.80)	0.08
FMI (kg/m^2^)	11.54 ± 3.58 (7.10–17.10)	10.53 ± 4.04 (5.70–18.60)	0.63
FFMI (kg/m^2^)	10.86 ± 1.23 (9.00–12.70)	13.30 ± 2.01 (9.60–15.40)	0.02
LMI (kg/m^2^)	10.20 ± 1.19 (8.50–12.10)	12.76 ± 1.87 (9.40–14.70)	0.01
ALMI (kg/m^2^)	3.84 ± 0.69 (3.14–4.78)	4.82 ± 0.81 (3.67–5.55)	0.03

*Kg, kilogram; BMI, body mass index; m^2^, square meter; FMI, fat mass index; FFMI, fat-free mass index; LMI, lean mass index; ALMI, appendicular lean mass index*.

*Summaries of individual data grouped according to sex. *p*-Value of the two sample *t*-test for the mean*.

### Muscle strength and physical function

Muscle strength was markedly reduced comparing with the normative values (van der Ploeg et al., 1991; Beenakker et al., 2001) (Table 2). Knee extension strength (KES) with a mean value of 60 N (range 15–132 N) was very weak comparing with healthy subjects in which it exceeded in both genders 250 N (van der Ploeg et al., 1991; Beenakker et al., 2001). Non-walkers had a significant lower (*p* ≈ 0.00) KES mean value as compared with walkers (22.5 N vs. 69.2 N). HGS was also very weak, and in all well below the *T*-score value less than −2 (20 kg in women and 30 kg in men). Again, there was a significant difference (*p* = 0.011) between mean HGS of non-walkers and walkers (2.9 kg vs. 13.0 kg), confirming that HGS is a good indicator of global muscle strength. The ratio between KES and body weight was in all well below (range 0.3–1.7 N/kg) the threshold (2.8 N/kg) in which performance on ambulatory tasks is compromised. Walking speed was below the threshold of 1.22 m/s, indicating a low mobility, in 10 patients (range 0–1, 11 m/s), and above it in 4 (range 1.25–1.72 m/s). % FVC was moderately (<70%) reduced in four, and severely reduced (<50%) in three patients (range 13–41%); non-walkers had a significantly lower (*p* = 0.03) % FVC as compared with walkers (39 vs. 72). Gender differences were significant only for FVC and % FVC.

**Table 2:** **Muscle strength and measured physical function**.

	Women (*n* = 7)	Men (*n* = 7)	*p*-value
HGS (kg)	9.69 ± 5.98 (1.00–18.50)	11.91 ± 6.35 (4.50–22.50)	0.51
KES (N)	62.86 ± 41.22 (15.00–131.00)	56.29 ± 40.67 (18.00–132.00)	0.77
KES/body weight (N/kg)	1.00 ± 0.57 (0.30–1.80)	0.90 ± 0.57 (0.40–1.70)	0.75
Gait speed (m/s)	1.01 ±0.73 (0.00–1.72)	0.81 ± 0.40 (0.00–1.25)	0.55
FVC (L)	1.74 ± 0.97 (0.46–2.77)	3.06 ± 0.62 (2.09–3.93)	0.01
% FVC	49.71 ± 29.12 (13.00–90.00)	79.43 ± 14.63 (53.00–90.00)	0.04

*HGS, handgrip strength; kg, kilogram; KES, knee extension strength; N, Newton; m, meter; s, second; FVC, forced vital capacity; L, liter*.

*Summaries of individual data grouped according to sex. *p*-value of the two sample *t*-test for the equality of the means*.

### Correlation between body composition, muscle strength, and physical function

Knee extension strength measures and gait speed showed moderate correlation coefficient significantly different from zero (Table 3). On the other hand, the coefficient between HGS and the physical function variables were all significant, showing a moderate to strong correlation (Table 3). The linear correlation coefficients between couples of possible explicative variables of muscularity showed values oscillating between 0.01 and 0.58 in absolute value. The indices of adiposity, total fat % and FMI, had non-significant linear correlation coefficient with all the muscle strength and physical function variables (Table 4). On the contrary, the indices of muscularity showed moderate positive values (0.40–0.58) when associated with FFMI and ALMI (Table 4); moreover, the HGS, FVC, and % FVC are significantly different from zero. The goodness of fit of the relationship between the predictor variables (BMI and ALMI) and the dependent variables (HGS, KES, % FVC, gait speed) was measured by the adjusted *R*^2^, which was always different from zero (Table 5). Its values are always highly significant and, therefore, suggest a good approximation of the suggested regression model.

**Table 3:** **Linear correlation coefficients between muscle strength (lines) and physical function (columns) variables**.

	Gait speed (m/s)	FVC (L)	% FVC
HGS (kg)	0.72 (0.00)	0.59 (0.03)	0.59 (0.03)
KES (N)	0.57 (0.03)	0.12 (0.68)	0.08 (0.77)
KES/body weight (N/kg)	0.55 (0.03)	0.05 (0.87)	0.11 (0.70)

*HGS, handgrip strength; kg, kilogram; KES, knee extension strength; N, Newton; m, meter; s, second; FVC, forced vital capacity; L, liter*.

*In parentheses, the *p*-value of the test for equality to zero of the correlation coefficient*.

**Table 4:** **Linear correlation coefficients between potential dependent (lines) and explanatory (columns) variables**.

	Total fat %	FMI	FFMI	ALMI
HGS (kg)	−0.21 (0.47)	0.03 (0.91)	0.58 (0.03)	0.51 (0.06)
KES (N)	0.20 (0.48)	0.36 (0.21)	0.43 (0.12)	0.48 (0.08)
Gait speed (m/s)	0.08 (0.79)	0.28 (0.34)	0.43 (0.13)	0.40 (0.15)
FVC (L)	−0.19 (0.51)	0.09 (0.75)	0.48 (0.08)	0.55 (0.04)
% FVC	−0.31 (0.28)	0.01 (0.96)	0.55 (0.04)	0.54 (0.05)

*HGS, handgrip strength; kg, kilogram; KES, knee extension strength; N, Newton; m, meter; s, second; FVC, forced vital capacity; L, liter; FMI, fat mass index; FFMI, fat-free mass index; ALMI, appendicular lean mass index*.

*In parentheses, the *p*-value of the test for equality to zero of the correlation coefficient*.

**Table 5:** **Linear models (*y*_i_ = β*x*_i_ +*e*_i_) of separate relative contribution of body mass and muscularity to muscle strength and physical function and the *p*-value of the test for equality to zero of the adjusted coefficient of linear determination**.

Dependent variables	Predictor variables	β	*R*^2^	*p*-Value
HGS (Kg)	BMI	0.46	0.78	0.00
	ALMI	2.53	0.82	0.00
KES (N)	BMI	2.62	0.75	0.00
	ALMI	14.05	0.75	0.00
% FVC	BMI	2.74	0.85	0.00
	ALMI	14.97	0.89	0.00
Gait speed (m/s)	BMI	0.04	0.75	0.00
	ALMI	0.21	0.75	0.00

**R*^2^, adjusted coefficient of linear determination; HGS, handgrip strength; kg, kilogram; KES, knee extension strength; N, Newton; FVC, forced vital capacity; m, meter; s, second; BMI, body mass index; ALMI, appendicular lean mass index*.

## Discussion

This study shows that adult MD patients can be classified according to the definition criteria of sarcopenia (Cruz-Jentoft et al., 2010). Our patients in spite of varying degree of impairment of muscle function, from mild to severe, all had “severe sarcopenia” that is the combination of low muscle mass, low muscle strength, and low physical performance (Cruz-Jentoft et al., 2010). The studied patients were also defined “sarcopenic obese” having in addition to a severe decrease in lean body mass an increase in FM (Baumgartner et al., 2004; Stenholm et al., 2008). ALMI was markedly reduced in all patients and correlated with the residual muscle strength in the arms and legs, and with physical performances determined by gait speed and FVC. In addition, ALMI and muscle strength were significantly reduced in non-walkers as compared to walkers.

A similar alteration in body composition, that is, decrease in lean body mass and increase in FM, was reported in patients with different types of MD (Skalsky et al., 2008, 2009; Pruna et al., 2011). Patients with facioscapulohumeral MD had higher fat tissue mass and lower lean tissue mass, despite similar BMI than controls (Skalsky et al., 2008). Patients with myotonic dystrophy had lower regional FFM index and higher fat mass index than controls (Pruna et al., 2011). However, in these studies, body composition was compared with age-matched, able-bodies controls, and not with the older adults impeding the recognition of sarcopenia.

Body composition, assessed by DXA, has started to be included in clinical trials as a primary or secondary endpoint in addition to the evaluation of muscle strength and function (Kissel et al., 2001; Merlini et al., 2008a; Vuillerot et al., 2014). A randomized, double-blind, placebo-controlled trial of albuterol in facioscapulohumeral dystrophy showed that although albuterol did not improve global strength or function, it did increase muscle mass assessed by DXA and improve some measures of strength (Kissel et al., 2001). The influence of a 2-year steroid treatment on body composition measured by DXA was evaluated in 29 DMD boys (Vuillerot et al., 2014). The 21 boys of the steroid group treatment improved significantly in body composition through a significant increase in lean tissue mass. In the steroid-naive patients, there were no significant increases in the lean tissue mass but deterioration in body composition was confirmed by a significant increase in the percentage of body FM (Vuillerot et al., 2014).

Loss of muscle mass and increased intramuscular fibrosis characterize both aging and MD. Research has shown that skeletal muscle wasting in aging and in muscular dystrophic share some pathophysiological mechanisms, including mitochondrial dysfunction (Irwin et al., 2003; Marzetti et al., 2013), increased apoptosis (Marzetti and Leeuwenburgh, 2006; Merlini et al., 2008a; Calvani et al., 2013), abnormal modulation of autophagy (Wohlgemuth et al., 2010; Merlini et al., 2014), decline in satellite cells (Thornell, 2011; Jiang et al., 2014), increased generation of reactive oxygen species (Doria et al., 2012; Canton et al., 2014), and modification of signaling and stress response pathways (Deldicque, 2013; Marzetti et al., 2013; Canton et al., 2014). Therefore, a standard evaluation of sarcopenia may be of benefit for both conditions: aging and MD.

Altogether, this study shows that aging sarcopenia criteria can be used in the evaluation of patients with MD providing relevant information in terms of residual muscle mass, muscle strength, and physical function, which may justify its broader implementation in future research, clinical trials, and clinical practice.

## Conflict of Interest Statement

The authors declare that the research was conducted in the absence of any commercial or financial relationships that could be construed as a potential conflict of interest.
